# Disinfection performance of mixed oxidant, NaClO, and Ca(ClO)_2_ in anaerobic effluents: effects on microbial quality, physicochemical properties, and trihalomethane formation

**DOI:** 10.1007/s11356-026-38060-9

**Published:** 2026-08-03

**Authors:** Alfredo Quirino Abreu Neto, Talita Marinho, Mario Takayuki Kato, Lourdinha Florencio

**Affiliations:** Department of Civil and Environmental Engineering, Laboratory of Environmental Sanitation, Federal University of Pernambuco, Recife, PE 50740-530 Brazil

**Keywords:** Ammonia oxidation, Chlorination, Conductivity, Disinfection byproducts, *Escherichia coli*, Total coliforms

## Abstract

The disinfectant action of the mixed oxidants (MIOXs) was compared with that of sodium and calcium hypochlorite (NaClO and Ca(ClO)_2_) in the post-treatment of anaerobic effluent. The following responses were evaluated: microbiological reduction (total coliforms and *Escherichia coli*); disinfection byproducts formation (total trihalomethanes—THM_4_, i.e., the sum of chloroform (TCM), bromodichloromethane (BDCM), dibromochloromethane (DBCM), and bromoform (TBM); and changes in physicochemical parameters: pH, temperature, redox potential, conductivity, chemical oxygen demand, ammonia nitrogen (N-NH_4_^+^) and ions: sodium, calcium, magnesium, potassium, chloride, chlorate, chlorite, nitrite, nitrate, sulfate, and phosphate. The experiment was conducted using a jar test system for 20 min at 100 rpm with a dosage of 2 mg of Cl_2_·L^−1^. Microbiological results indicated greater efficiency in the removal of total coliforms for MIOX, with results showing a log reduction from 6.23 log_10_ MPN per 100 mL to 1.68 ± 0.13, 1.94 ± 0.08, and 2.53 ± 0.39 log_10_ MPN per 100 mL for Ca(ClO)_2_, NaClO, and MIOX, respectively. THM_4_ formation was predominantly TCM, with levels below the limit of detection for DBCM, BDCM, or TBM. The increases in electrical conductivity stood out among the physicochemical parameters, being more pronounced for MIOX. This result suggested that MIOX may have promoted greater ionic transport in the aqueous matrix, thereby enhancing disinfection. MIOX also demonstrated greater efficiency in oxidizing nitrogen compounds. This study suggests that mixed oxidants can exert a combined effect on the removal of nitrogen compounds, thereby improving the treatment of anaerobic wastewater containing N-NH_4_^+^.

**Supplementary Information:**

The online version contains supplementary material available at 10.1007/s11356-026-38060-9.

## Introduction

Anaerobic treatment processes are widely used because they reduce energy costs, minimize sludge production, and enable resource recovery (Dutta et al. 2018; Zhang and Li 2019; Phuc-HanhTran et al. 2024). One advantage of anaerobic treatment compared to aerobic processes is its ability to handle wastewater with high organic loads, achieving chemical oxygen demand (COD) removal efficiencies above 90% and allowing for the use of more compact treatment units (Hutnan et al. 1999; Wang et al. 2022a). However, biological processes in wastewater treatment are not sufficiently efficient at removing pathogens. Typically, two main systems are used for domestic effluent disinfection: maturation ponds, which require large land areas, and chemical oxidation, which only requires a compact area and simple equipment (Corporation 2010; Dias et al. 2014).

Among chemical oxidants, chlorine is the main technology used for wastewater disinfection. However, treatment plants are constantly seeking new technologies that offer greater operational and environmental safety, thereby improving processes (Mazhar et al. 2020). In this context, electrochemical disinfection through on-site generation of sodium hypochlorite (NaClO) is gaining significant acceptance in the water industry (Casson and Bess 2003; AWWA 2015). On-site production of NaClO via electrolysis is an alternative for water disinfection (Casson and Bess 2003; Ponzano 2007). However, few studies have investigated reactions and byproducts generated during wastewater disinfection, underscoring that the impacts of this oxidizing agent remain poorly understood, including aspects related to health, costs, and environmental risks.

The on-site production of NaClO has emerged as an innovative and safe alternative, offering benefits such as reduced chemical volume, enhanced worker safety, and the elimination of liabilities related to transportation and safety management (Sousa Filho et al. 2022). This technology results from the electrolysis of a saline solution composed of sodium chloride (NaCl), which generates hypochlorous acid (HOCl) in equilibrium with the hypochlorite ion (OCl⁻), along with other chlorine species (Wu et al. 2019; Scialdone et al. 2021; Choi et al. 2022) The solution produced, known as mixed oxidant (MIOX), includes dichlorine, chlorine dioxide, ozone, hydrogen peroxide, and NaClO (Wu et al. 2019; Scialdone et al. 2021). As such, MIOX acts as a powerful oxidant in the effective inactivation of pathogenic microorganisms present in (Venczel et al. 1997; Estrela et al. 2002; Fukuzaki 2006; Wu et al. 2019; Bae et al. 2023).

Although chlorination treatment reduces the risk of contamination by pathogenic microorganisms, its use can lead to the formation of undesirable byproducts during disinfection. These byproducts are formed through reactions between natural organic matter (predominantly humic and fulvic acids) and halogenated compounds (Gallard and Von Gunten 2002; Mazhar et al. 2020). Trihalomethanes (THMs) are formed due to the presence of chlorine and/or bromine (Srivastav et al. 2020). THMs are formed by substituting three hydrogen atoms in methane (CH4) with halogenated compounds, chlorine and bromine, or their combinations (Mazhar et al. 2020; Srivastav et al. 2020). This group is limited to the four most commonly observed species: trichloromethane (TCM), bromodichloromethane (BDCM), dibromochloromethane (DBCM), and tribromomethane (TBM), collectively known as THM_4_ (Thokchom et al. 2020).

In the context of wastewater treatment, recent studies have explored the use of MIOX solution (Abreu Neto et al. 2025; Quirino et al. Abreu Neto et al. (2025) reported low trihalomethane (THM) formation, even under breakpoint chlorination conditions, during treatment of anaerobic effluent. Additionally, the use of MIOX has been investigated for its potential to remove other organic compounds from anaerobic wastewater (Quirino et al. 2025). Quirino et al. (2025) observed that when treating linear alkylbenzene sulfonate (LAS), MIOX outperformed NaClO, achieving a 14.5% removal rate compared to 8% with NaClO. The findings emphasize MIOX’s potential as a viable alternative oxidizing agent, owing to the combined action of electrochemically generated oxidants. The mineralization potential of emerging contaminants increases when multiple oxidizing agents are combined (Preethi et al. 2023).

The presence of THMs in the environment is associated with risks to human health, as they can be ingested or absorbed through the skin when present in water, especially when treated water is used for consumption. Among the health issues linked to exposure to these compounds are an increased risk of cancer and associations with comorbidities such as liver damage and type II diabetes (Andra et al. 2014; Siddique et al. 2015; Makris et al. 2016; Chowdhury et al. 2020). Therefore, monitoring these substances serves as a biomarker of exposure to disinfection byproducts, contributing to the prevention of health disorders such as genotoxicity, mutagenicity, and alterations in the central nervous system (Andra et al. 2014; Makris et al. 2016; Srivastav et al. 2020).

Other parameters are also equally important to monitor. These parameters not only help assess water quality but also directly influence the formation of disinfection byproducts, such as ionic concentrations. Monitoring ions in wastewater is essential for understanding the level of contamination and the chemical variations present, as ions such as sodium, potassium, calcium, and ammonium can indicate significant changes in water composition (Le Bonté et al. 2008). Ionic analysis, therefore, contributes to understanding pollution patterns and the effectiveness of treatment processes.

In addition, monitoring ion concentrations is key to understanding the mechanisms underlying disinfection with chlorinated compounds. Metallic ions, for example, can interfere with the formation of byproducts such as THMs (Navalon et al. 2009; Sharma et al. 2017). Zhang et al. (2019) investigated how different ionic species influence the formation of disinfection byproducts (DBPs) during chlorination and chloramination processes (Zhang et al. 2019). The authors observed that cations such as calcium (Ca^2+^) and trivalent aluminum (Al^3+^) significantly impact this formation. Ca^2+^, for instance, can reduce THM formation by strongly binding to natural organic matter (NOM) through electrostatic interactions with its ionized groups. In contrast, anions such as nitrate (NO_3_^−^) and sulfate (SO_4_^2−^) showed minimal effects on DBP formation. Therefore, assessing the presence of these ions is also a crucial parameter, as they directly influence one of the central variables analyzed in this study: THM formation.

Despite advances in the application of hypochlorite in water treatment, there is still a need for a better understanding of its efficiency in removing pathogenic microorganisms, the formation of chlorination byproducts such as THMs, and the influence of ions on the generation of these compounds when MIOXs are applied. In this context, the research comparatively evaluates the oxidative performance of NaClO, Ca(ClO)_2_, and MIOX hypochlorites in anaerobically sourced domestic wastewater, focusing on three aspects: the microbiological reduction of *E. coli* and total coliforms; the formation of disinfection byproducts, specifically THM_4_ (TCM, BDCM, DBCM, and TBM); and the physicochemical monitoring parameters, including pH, ORP, conductivity, COD, N-NH_4_^+^, as well as the quantification of cations: sodium (Na^+^), Ca^2+^, magnesium (Mg^2+^), potassium (K^+^) and anions: chloride (Cl^−^), chlorate (ClO_3_^−^), chlorite (ClO_2_^−^), nitrite (NO_2_^−^), NO_3_^−^, SO_4_^2−^, and phosphate (PO_4_^3−^).

## Material and Methods

### Chemicals

The analytical-grade compounds used were acquired from Sigma-Aldrich: HPLC-grade methanol, tert-butyl methyl ether, sodium sulfate, fluorobenzene solution (2000 µL·L^−1^), and EPA 501/601 trihalomethanes calibration mix. The THMs mixture consisted of four environmental pollutants: bromoform (TBM), bromodichloromethane (BDCM), dibromochloromethane (DBCM), and chloroform (TCM). Additional reagents used in the experiments were magnesium chloride hexahydrate (MgCl_2_·6H_2_O), ammonium chloride (NH_4_Cl), potassium chloride (KCl), NaCl, calcium chloride (CaCl_2_·2H_2_O), sodium chlorate (NaClO_3_), sodium chlorite (NaClO_2_), monopotassium phosphate (KH_2_PO_4_), potassium sulfate (K_2_SO_4_), sodium nitrate (NaNO_3_), ascorbic acid (C_6_H_8_O_6_), sodium thiosulfate (Na_2_S_2_O_3_), sodium carbonate (Na_2_CO_3_), sodium bicarbonate (NaHCO_3_), and sulfuric acid (H_2_SO_4_). The reagents NaClO (5% active chlorine w/w) and Ca(ClO)_2_ (65% active hypochlorite) were commercially obtained. The water used was purified using the Milli-Q® reference system from Merck Millipore. The resistivity of the deionized water used was 18 MΩ·cm^−1^.

### Wastewater sampling

The wastewater sample was collected from the effluent of an up-flow anaerobic sludge blanket reactor (UASB) at a domestic wastewater treatment plant in Recife-PE, Brazil. It was immediately taken to the laboratory and filtered using a downflow filter made of gravel (30 cm) and activated carbon (30 cm) at a water flow rate of approximately 7 mL·s^−1^. It was then stored at 4 ºC.

### Mixed oxidant (MIOX)

MIOX solution was produced in a static generator coupled to an electrolytic cell; the equipment had a capacity of 150 L of MIOX at a dosage of 6 kg sodium chloride (NaCl) and a reaction time of 24 h. The equipment was manufactured by Hidrogeron® (Paraná, Brazil).

### Experimental design: hypochlorite disinfection

The experiments were performed with a sample volume of 1.5 L in a jar test agitation system with a total volume of 2 L. The chlorination experiment was performed in a well-mixed batch reactor with an agitator of 100 rpm, corresponding to a velocity gradient of 100 s^−1^.

### Determination of contact time

Initially, the disinfectant efficacy of Ca(ClO)_2_, NaClO, and the MIOX solution was evaluated based on total coliform and *Escherichia coli* (*E. coli*) concentrations. The objective was to define the optimal contact time and working dosage for disinfection. At this stage, the influence of the combinations of contact times (10, 15, and 20 min) and dosages (1.0, 1.5, and 2.0 mg·L^−1^) on disinfection was observed. After each chlorination experiment, dechlorination was performed using Na_2_S_2_O_3_.

### Disinfection treatment

At this stage of the experiment, the following steps were performed in triplicate. During this part of the experimental design, the disinfection of pathogenic microorganisms, the formation of disinfection byproducts such as THM_4_, and the influence of the oxidants, Ca(ClO)_2_, NaClO, and the MIOX solution were evaluated. All experiments were conducted with a contact time of 20 min and a dosage of 2 mg·L^−1^, followed by dechlorination with Na_2_S_2_O_3_ and C_6_H_8_O_6_. The dechlorination strategy using C_6_H_8_O_6_ was adopted to avoid interference in the quantification of Na^+^, and SO_4_^2−^ ions in chromatographic analyses. After all experiments, the samples were preserved according to the Standard Methods (APHA 2017).

### Analytical methods

A wide range of parameters were evaluated; they are chemical oxygen demand—COD (5220 C), ammonia nitrogen—N-NH_4_^+^ (4500 B), residual free chlorine, combined residual chlorine (CRC) as mono-, di-, and tri- chloramine (NH_2_Cl, NHCl_2_, and NCl_3_, respectively), and total residual chlorine (TRC) (4500 G). The microbiological determination was performed using the defined chromogenic substrate method ONPG-MUG, Colilert® kit (IDEXX Laboratories) in combination with the Quanti-Tray/2000 for the detection of total coliforms and *E. coli*, according to Standard Methods 9223 B (APHA 2017). Prior to analysis, the samples were subjected to serial dilutions in sterile buffered water, with dilution factors ranging from 10^–2^ to 10^–5^, depending on the expected microbial load. Aliquots of the diluted samples were analyzed, and the most probable number (MPN) values were estimated from the number of positive wells, corrected by the corresponding dilution factor, and expressed as MPN per 100 mL. Microbial reduction was subsequently calculated and expressed as log_10_ reduction based on the difference between initial and final concentrations. These parameters were evaluated according to the Standard Methods (APHA 2017). Hydrogenionic potential (pH), temperature, redox potential (ORP), and conductivity were measured using a multiparameter probe (Hach HQ40d, USA).

The ion determinations were performed using ion chromatography (ICS-2100, Dionex, USA). For anion determination, a Dionex IonPac AS23 analytical column (2 × 250 mm), equipped with a guard column, was used with a carbonate/bicarbonate mobile phase (4.5 mM Na_2_CO_3_ and 0.8 mM NaHCO_3_) at a flow rate of 0.25 mL·min^−1^. The column oven temperature was maintained at 30 °C, and the suppressor current was set at 7 mA. The injection volume was 25 µL. Analyses were conducted in accordance with APHA (2017), Method 4110 B. The method detection limits (LODs) ranged from 2.0 to 18 µg·L^−1^, and the limits of quantification (LOQs) were estimated at 3–10 times the corresponding LODs. For cation determination, a Dionex IonPac CS12A column (8 µm, 2 × 250 mm) was used, with an 11 mM H_2_SO_4_ mobile phase, a 2 mm CSRS conductivity suppressor, a flow rate of 0.38 mL·min^−1^, and an injection volume of 5 µL. LOD and LOQ were experimentally determined. The LODs ranged from 0.01 to 1.44 mg·L^−1^, while the LOQs ranged from 0.03 to 4.73 mg·L^−1^ (Silva et al. 2026). The quantified ions were Na^+^, Ca^2+^, Mg^2+^, K^+^, Cl^−^, ClO_3_^−^, ClO_2_^−^, NO_2_^−^, NO_3_^−^, SO_4_^2−^, and PO_4_^3−^. Calibration curves ranged from 1–60 mg·L^−1^ and 50–300 mg·L^−1^.

THM_4_ concentrations were measured by an analytical liquid–liquid extraction-gas chromatography-mass spectrometry (GC–MS) (GC: model 7890 A, Agilent, U.S.; MS model 5975 C, Agilent, U.S.) using an SLB® 5MS column (30 m × 0.25 mm × 0.25 µm, Sulpeco® 28,471 – U with helium (99.99% purity) as carrier gas at 1.0 mL min^−1^ (Franco et al. 2018). Samples were injected in splitless mode (2 µL), with the oven programmed from 35 °C (1 min) to 40 °C at 1 °C min^−1^ (hold 1 min), then to 200 °C at 10 °C·min^−1^ (hold 1 min); injector temperature was 200 °C, trap 180 °C, and transfer line 200 °C. THMs were extracted immediately from disinfected wastewater using MTBE. THM_4_ was calculated as the sum of TCM, TBM, DCBM, and BDCM. Calibration curves ranged from 40 to 400 µg·L^−1^ for TCM and from 1 to 30 µg·L^−1^ for TBM, DCBM, and BDCM, with high linearity. Results were compared with a 50 µL internal standard of fluorobenzene (200 µg·L^−1^). The LOD/LOQ values of the method for measuring THMs were: TCM (0.13/0.40 µg·L^−1^), BDCM and DBCM (0.02/0.07 µg·L^−1^), and TBM (0.01/0.05 µg·L^−1^).

### Statistical analysis

Descriptive statistics and correlation analyses were performed using GraphPad Prism version 9. Differences in N-NH_4_^+^ removal were assessed by one-way ANOVA followed by Tukey’s post hoc test. Log_10_ reductions in total coliforms were analyzed using the Kruskal–Wallis test, followed by Dunn’s post hoc test with Bonferroni correction. Statistical significance was set at *p* < 0.05.

## Results and discussion

### Determination of contact time and working dosage

The parameters used in the assays to determine contact time and working dosage were defined by combining different contact times (10, 15, and 20 min) with disinfectant dosages (1.0, 1.5, and 2.0 mg·L^−1^), while simultaneously evaluating the reduction of total coliforms and *E. coli* in anaerobic effluents treated with Ca(ClO)_2_, NaClO, and the MIOX solution. The complete information from the preliminary assays is provided in the Supplementary Information (SI). To standardize and ensure reliability across the tests, a dosage of 2.0 mg·L^−1^ and a contact time of 20 min were adopted as the experimental conditions.

### Evaluation of physicochemical parameters

Table 1 compares the different physicochemical parameters monitored for the sample before and after oxidative treatment with commercial hypochlorites: NaClO and Ca(ClO)_2_, and the MIOX solution. Table 1 presents the following parameters: pH, ORP, conductivity, COD, N-NH_4_^+^, and ions: Na^+^, Ca^2+^, Mg^2+^, K^+^, Cl^−^, ClO_3_^−^, ClO_2_^−^, NO_2_^−^, NO_3_^−^, SO_4_^2−^, and PO_4_^3−^.

**Table 1:** Physicochemical parameters evaluated in the pre- and post-treatment with NaClO, Ca(ClO)_2_, and MIOX

Parameters	Pre-treatment	Post-treatment		
	Raw sample	MIOX	NaClO	Ca(ClO)_2_
Conductivity (μS·cm^−1^)	0.2 ± 0.0	810 ± 9	587 ± 58	597 ± 0.0
ORP (mV)	55.2 ± 0.5	64.9 ± 4.0	77.7 ± 0.4	63.2 ± 3.5
pH	6.30 ± 0.10	6.92 ± 0.01	6.82 ± 0.06	6.75 ± 0.01
N-NH_4_^+^ (mg·L^−1^)	21.4 ± 0.1	18.2 ± 0.2	21.2 ± 0.0	21.4 ± 0.1
COD (mg·L^−1^)	128 ± 0	121 ± 18	121 ± 16	123 ± 12
Na^+^ (mg·L^−1^)	50.6 ± 0.1	91.2 ± 16.2	62.5 ± 1.6	56.6 ± 0.5
K^+^ (mg·L^−1^)	10.9 ± 0.1	9.9 ± 1.0	10.3 ± 0.9	10.2 ± 0.9
Mg^2+^ (mg·L^−1^)	6.7 ± 0.0	5.6 ± 1.0	6.2 ± 0.1	6.1 ± 0.0
Ca^2+^ (mg·L^−1^)	18.3 ± 0.0	16.4 ± 2.4	17.8 ± 0.2	18.7 ± 0.0
NO_2_^−^ (mg·L^−1^)	34.4 ± 0.2	36.2 ± 0.2	37.4 ± 0.1	37.6 ± 0.4
NO_3_^−^ (mg·L^−1^)	3.8 ± 0.1	7.0 ± 0.0	7.0 ± 0.1	6.7 ± 0.1
PO_4_^3−^ (mg·L^−1^)	21.7 ± 0.0	19.4 ± 0.4	19.3 ± 0.0	19.3 ± 0.5
SO_4_^2−^ (mg·L^−1^)	146.6 ± 0.6	61.7 ± 0.4	61.9 ± 0.4	62.0 ± 0.3
Cl^−^ (mg·L^−1^)	15.5 ± 0.4	126.2 ± 0.6	73.7 ± 0.1	67.2 ± 0.2
ClO_3_^−^ (mg·L^−1^)	3.8 ± 0.0	3.6 ± 0.0	3.6 ± 0.0	3.7 ± 0.0
ClO_2_^−^ (mg·L^−1^)	< LOD*	< LOD*	< LOD*	< LOD*

* < LOD = Below detection limit

The experiment was conducted at a constant temperature of 21.8 ± 0.1 **°**C for the three oxidizing agents studied: NaClO, Ca(ClO)_2_, and MIOX. As shown in Table 1, ORP increased from 55 mV to 77.7, 63.2, and 64.9 mV, respectively, due to the redox reactions between chlorine and ammonia. (Bergendahl and Stevens 2005). In these reactions, the variation in ORP is related to electron transfer: chlorine is oxidized and ammonia is reduced, forming different chloramine species. This process is common in wastewater. Thus, these reactions alter the redox balance of the solution, increasing the ORP due to the presence of these oxidizing or reducing species. ORP is therefore an important tool for monitoring disinfection by chlorinated species such as NaClO, Ca(ClO)_2_, and MIOX (Kim and Hensley 1997; Bergendahl and Stevens 2005).

Regarding pH variation, Table 1 shows increase from 6.30 to 6.82, 6.75, and 6.92 for the respective oxidants NaClO, Ca(ClO)_2_, and MIOX. This occurs because hypochlorite, when dissolved in water, generates OH⁻ ions, which raise the pH. The OH⁻ ions make the solution more alkaline, creating an environment where hypochlorite can act as an effective disinfectant. However, the results indicated that the pH after the experiment remained below 7. Therefore, it can be concluded that hypochlorous acid (HOCl) was the predominant disinfectant species, since pH values below its pKa < 7.5 favor the undissociated molecular form (Fukuzaki 2006).

The effects of NaClO, Ca(ClO)2, and MIOX on COD removal were investigated. The COD remained virtually constant across all hypochlorites used, ranging from 128 mg·L^−1^ to 121, 123, and 121 mg·L^−1^ for NaClO, Ca(ClO)_2_, and MIOX, respectively. These slight reductions are within the analytical uncertainty of the COD method (approximately 5.6%, according to APHA 5220 C) and therefore, do not indicate a significant effect of chlorination on organic load. Chlorine doses below 10 mg·L^−1^ do not significantly affect COD, as demonstrated by El-Rehaili (1995), who reported only minor variations within this concentration range (El-Rehaili 1995). Therefore, the low dosage applied in this study (2 mg·L^−1^) is expected to minimize impacts on effluent quality.

The initial N-NH_4_^**+**^ concentration of 21.4 mg·L^−1^ after the chlorination process decreased by 21.2, 21.4 and 18.2 mg·L^−1^ when treated with NaClO, Ca(ClO)_2_, and MIOX, respectively. A one-way analysis of variance (ANOVA) revealed highly significant differences among the treatment groups, indicating that at least one treatment had a statistically distinct effect. Subsequent Tukey’s post hoc test showed that the MIOX treatment achieved significantly higher N-NH_4_^+^ removal rates compared to those of NaClO and Ca(ClO)_2_ (*p* < 0.05). This reduction can be attributed to the mixture of multiple oxidants present in the MIOX composition. It is important to note that the free radical HO• did not directly oxidize ammonia in the absence of Cl^−^ ions, while Cl•, ClO•, and free chlorine showed high oxidation capacity, with nitrogen selectivity above 90% (Zhang et al. 2018; Zheng et al. 2020; Li et al. 2023). According to Gupta et al. (1972), based on the evaluation of the reaction kinetics between ammonia and chlorine, the hypochlorite ion (OCl^−^) is the reactive species that interacts with NH_4_^+^ (Gupta et al. 1972). This reaction, which occurs between ions of opposite charge, is favored at pH values above 5, as observed in all disinfection processes studied (Table 1), and may explain its rapid kinetics relative to predictions based on collision theory.

The initial Cl^−^ concentration was 15.5 mg·L^−1^, and it increased to 73.7, 67.2, and 126.2 mg·L^−1^, after disinfection with NaClO, Ca(ClO)_2_, and MIOX. This increase is due to the oxidation of ammonia by chlorine, which produces chloride ions as byproducts according to the reaction (1).

1 $$2\,{ NH}_{3}+3 \,{Cl}_{2} \to {N}_{2}+6 \,HCl$$

As a consequence of the increased Cl^−^ concentration, the conductivity increased due to the higher salt concentration in the experiment. This raised the conductivity from 0.2 μS·cm^−1^ to 587, 597, and 810 μS·cm^−1^, corresponding to NaClO, Ca(ClO)_2_, and MIOX, respectively. The higher conductivity observed for MIOX (Table 1) suggests a greater contribution of dissolved ionic species, which may enhance ionic mobility in the aqueous matrix (Le Bonté et al. 2008). This effect is relevant for disinfection processes, as the presence and movement of ions can influence the effectiveness of oxidative treatments in aqueous matrices (Vincent et al. 2016).

It is important to highlight that not only Cl^−^ plays a role in the dynamics of oxidative processes. Other ionic species, such as ClO_3_^−^, ClO_2_^−^, NO_2_^−^, and NO_3_^−^, can also be influenced by, or even influence, the treatment with HOCl obtained from chlorination processes. It is emphasized that although it is expected that most of the ammonia will be converted to nitrogen gas (N_2_) as demonstrated in reaction (1), other intermediate nitrogenous byproducts, such as NO_2_^−^ and NO_3_^−^, may be generated in this process, as shown in reaction (2) (Zhang et al. 2018).

2 $${NH}_4^++4\,HOCI\rightarrow{NO}_{3^-}+H_2O+6\,H^++4\,{Cl}^-$$

Table 1 shows the concentration of 21.4 mg·L^−1^ of N-NH_4_^+^, one of the precursors for the formation of NO_3_^−^ and NO_2_^−^. N-NH_4_^+^ is a nitrogenous compound that naturally forms part of the composition of compounds found in domestic wastewater (Dong et al. 2019). NO_3_^−^ concentration increased from 3.8 mg·L^−1^ to 7.0, 6.7, and 7.0 mg·L^−1^, while NO_2_^−^ concentration remained practically constant, with only a slight rise from 34.4 mg·L^−1^ to 37.4, 37.6, and 36.2 mg·L^−1^; these changes correspond to disinfection with NaClO, Ca(ClO)_2_, and MIOX, respectively (Table 1).

According to Margerum et al. (1994), the formation of NO_3_^−^ from NO_2_^−^ and chloramine involves several complex reactions with reactive intermediates, such as chlorine nitrite, NO_2_Cl, which are favored in acidic conditions due to protonation. The protonation of chloramine directly influences its ability to transfer Cl^−^, while factors such as pH and concentration are crucial for understanding the kinetics and mechanisms of these reactions in aquatic environments, especially in wastewater treatment (Margerum et al. 1994). Although Margerum et al. (1994) do not specify an ideal pH, chloramine reactions with NO_2_^−^ are generally more efficient at pH values lower than 7, where H⁺ ion activity promotes NO_2_Cl formation. At higher pH, chloramine may decompose, reducing the efficiency of NO_2_^−^ oxidation to NO_3_^−^. In the present study, the pH values of the oxidizing agents were 6.82, 6.75, and 6.92 for NaClO, Ca(ClO)2, and MIOX, respectively, providing favorable conditions for NO_3_^−^ formation from NO_2_^−^.

In Table 1, no significant variation was observed in the concentrations of chlorinated intermediate byproducts, such as ClO_2_^−^ and ClO_3_^−^. In the experiment, ClO_2_^−^ was not detected. Busch et al. (2019) discussed the formation of ClO_2_^−^ as an intermediate in the decomposition reactions of HOCl. According to the authors, ClO_2_^−^ can form via a concerted reaction in which OCl^−^ attacks the chlorine atom of another OCl^−^, releasing Cl^−^. ClO_2_^−^ formation occurs, preferentially under alkaline conditions (pH ≥ 9), where OCl^−^ is available and thermodynamically favored. The reaction leading to the formation of ClO_2_^−^ has a relatively high activation barrier, making this pathway relevant only under specific conditions. Thus, the formation of ClO_2_^−^ is closely associated with reactions occurring at an alkaline pH, where OCl^−^ is the main available reagent. In the present experiment, the pH variation was below 7, which did not favor ClO_2_^−^ formation. However, ClO_3_^−^ concentrations were quantified and showed no significant variations after disinfection with NaClO, Ca(ClO)_2_, and MIOX. The initial concentration of 3.8 mg·L^−1^ decreased slightly to 3.6–3.7 mg·L^−1^ after the treatments, indicating that no significant degradation of ClO_3_^−^ occurred under the experimental conditions of the study.

SO_4_^2−^ concentration decreased markedly after disinfection. The initial concentration of 146.6 mg·L^−1^ decreased to 61.9, 62.0, and 61.7 mg·L^−1^ after treatment with NaClO, Ca(ClO)_2_, and MIOX, respectively. Monitoring changes in SO_4_^2−^ concentration is useful for tracking undesirable byproducts such as sulfides, which are highly toxic to the environment (Bagarinao 1992), although SO_4_^2−^ does not directly influence DBPs formation (Zhang et al. 2019). PO_4_^3−^ concentrations were also quantified and showed a slight reduction. The initial concentration of 21.7 mg·L^−1^ decreased to 19.3, 19.3, and 19.4 mg·L^−1^, after disinfection with NaClO, Ca(ClO)_2_, and MIOX, respectively. The oxidation of this ion shows the same behavior for the studied hypochlorites.

For the cations Na^+^, K^+^, Mg^2+^, and Ca^2+^, no substantial variation was observed in K^+^ and Mg^2+^ concentrations. However, Na^+^ concentrations increased after the addition of NaClO and MIOX, rising from 50.6 mg·L^−1^ to 62.5 and 91.2 mg·L^−1^, respectively. The increase in Na⁺ concentration after treatment with MIOX is likely associated with residual unconverted NaCl during electrolysis and the release of Na⁺ as a byproduct of the MIOX generation process. Consequently, future studies may explore additional treatment steps, such as ion exchange or reverse osmosis, to reduce residual Na⁺ concentrations. An increase, though slight, was also observed in Ca^2+^ concentration when Ca(ClO)_2_ was used, from 18.3 mg·L^−1^ before treatment to 18.7 mg·L^−1^ after treatment. The presence of Ca^2+^ is associated with a reduction in the formation of trihalomethanes (THMs) (Zhang et al. 2019), as well as the presence of Mg^2+^, since they can bind to dissolved organic matter through carboxyl groups, which affects the formation of THMs during the chlorination process (Navalon et al. 2009; Sharma et al. 2017; Zhang et al. 2019).

### Effect of disinfection intervention on microbiological reduction

**Fig. 1:**
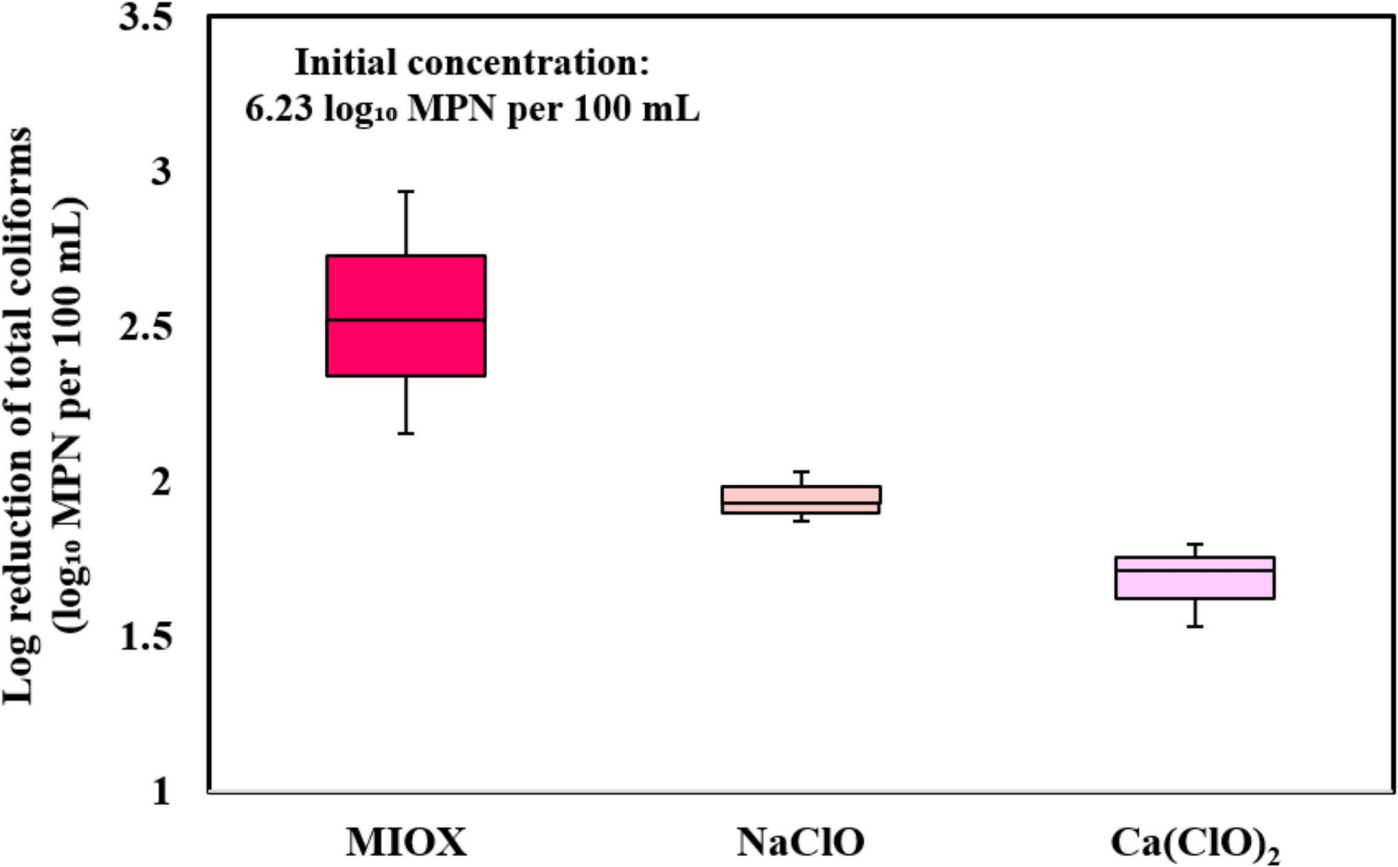
Log_10_ reduction of total coliforms (MPN per 100 mL) after disinfection with Ca(ClO)_2_, NaClO, and MIOX. Box plots represent median and interquartile range (*n* = 3)

Bacteriological quantification was performed for total coliforms and *E. coli*. The results of the total coliform concentration after wastewater disinfection with the hypochlorites Ca(ClO)_2_, NaClO, and MIOX are presented in Fig. 1.

The results shown in Fig. 1 present the log_10_ reduction of total coliforms (MPN per 100 mL) after disinfection using MIOX, NaClO and Ca(ClO)_2_. The initial concentration of total coliforms was 6.23 log_10_ MPN per 100 mL. Figure 1 shows that the oxidative action of the applied hypochlorites had a different effect on the inactivation of total coliforms. Among the evaluated treatments, MIOX achieved the highest log_10_ reduction of total coliforms (2.53 ± 0.39 log_10_ MPN per 100 mL), indicating greater disinfection efficiency, while NaClO (1.94 ± 0.08 log_10_ MPN per 100 mL) showed intermediate performance and Ca(ClO)_2_ the lowest reduction (1.68 ± 0.13 log_10_ MPN per 100 mL). Statistical analyses were performed to evaluate whether there were significant differences among the treatments. The Kruskal–Wallis test indicated significant differences between the groups (Kruskal–Wallis H = [0.021], *p* < 0.05), followed by Dunn’s post hoc test with Bonferroni correction (*p* < 0.05; n = 3 replicates per treatment). MIOX showed a significantly higher total coliform reduction compared to Ca(ClO)_2_, while no significant differences were observed between MIOX and NaClO or between NaClO and Ca(ClO)_2_.

The initial concentration of *E. coli* was 5.91 log_10_ MPN per 100 mL. After disinfection with Ca(ClO)_2_, NaClO, and MIOX, the *E. coli* concentrations were reduced to below the detection limit (< 1 MPN per 100 mL) for all chlorinated agents tested. This indicates that all the applied hypochlorites are effective in removing this microorganism. This result supports the study by Owoseni and colleagues (2017), which examined the survival of *E. coli* at different chlorine concentrations. The authors demonstrated high efficiency in *E. coli* inactivation over time, reaching an optimal chlorine dose of 1.5 mg·L^−1^. It is worth noting that, in the present study, the applied dosage was 2 mg Cl_2_·L^−1^ (Owoseni et al. 2017).

Chlorine compounds, such as Ca(ClO)_2_ and NaClO, when added to water, react to form hypochlorous acid (HOCl), which dissociates into OCl^−^ and H^+^ (Gonçalves et al. 2003; Gray et al. 2013). HOCl and OCl^−^ are powerful chemical species that can react with proteins, sulfur-containing amino acids, nucleotides, and lipids. These oxidizing agents are known to cause damage to the cellular morphology of microorganisms, including bacteria such as *E. coli* and total coliforms, leading to cellular inactivation (Bridges et al. 2022). HOCl can cross the cell membrane because it lacks a charge, unlike OCl^−^, which, being charged, cannot penetrate intact cell membranes (Fukuzaki 2006). However, Ersoy and colleagues conducted a comparative evaluation in 2019 of the disinfection efficiency of NaClO, ClO2, and MIOX mechanisms for inactivating the bacterium *Enterococcus faecalis* (*E. faecalis*).

They aimed to examine cellular viability, membrane integrity, and cellular components to reveal the underlying mechanisms of the disinfection process. The results showed that MIOX was the most effective oxidant for bacterial inactivation in a short period of time compared to the other oxidants. These results may explain the behavior observed in Fig. 1. The results obtained by those authors showed 95.4% bacterial elimination in 1 min when MIOX was applied, while NaOCl and ClO2 achieved 73.9% and 72.6% bacterial inactivation after 20 min, respectively.

According to the same authors, the diffusion of chlorine species in the intracellular area inactivated the cell in the same way when NaClO, ClO_2_, and MIOX were applied. This same similarity in the disinfection process was observed by Choi et al. (2022) when comparing MIOX with gaseous chlorine. However, MIOX was shown to damage the membrane more quickly, as indicated by increased conductivity and lipid peroxidation within 30 s (Ersoy et al. 2019). According to these authors, all disinfectants inactivated bacteria by disrupting membranes, causing ionic imbalances, and causing cellular damage (Ersoy et al. 2019). Thus, the key to disinfection efficiency lies in the combined action of the MIOXs.

According to Bonetta et al. (2021), disinfection with NaClO is the most effective treatment in terms of reducing indicator microorganisms of effluent contamination (Bonetta et al. 2021). However, the results of the present study update that information, indicating that MIOX is effective against total coliforms and *E. coli*. Thus, it can be inferred that microbiological deactivation using MIOX is more efficient at removing microorganisms in anaerobic wastewater. MIOX proved to be a technology with the potential to significantly reduce the costs of microbiological disinfection compared to Ca(ClO)_2_ and NaClO. Another advantage of MIOX may be its ability to control biofilm formation (Choi et al. 2022; Oliveira et al. 2024).

The fraction of hypochlorous acid (HOCl) was estimated using the Henderson–Hasselbalch equation (pKa ≈ 7.5 at 21.8 °C), and the pH values applied for each oxidizing agent are presented in Table 1. The calculated HOCl fractions were 79.2% for MIOX, 82.7% for NaClO, and 84.9% for Ca(ClO)_2_, indicating that HOCl was the predominant species under all experimental conditions. Since HOCl is the most effective disinfectant due to its ability to penetrate microbial cell membranes, these results confirm that disinfection was favored across all treatments. However, despite presenting a slightly lower HOCl fraction, MIOX achieved higher microbial inactivation, suggesting that additional oxidant species contributed to its enhanced disinfection performance as shown in Fig. 1. Cho and colleagues (2010), when evaluating the action of different oxidant agents on *E. coli* inactivation, observed that strong oxidants, such as ozone, caused greater damage to the cell surface, while damage to the internal components of the cell was more pronounced with weaker oxidants like free chlorine (Cho et al. 2010).

### Formation of trihalomethanes

**Fig. 2:**
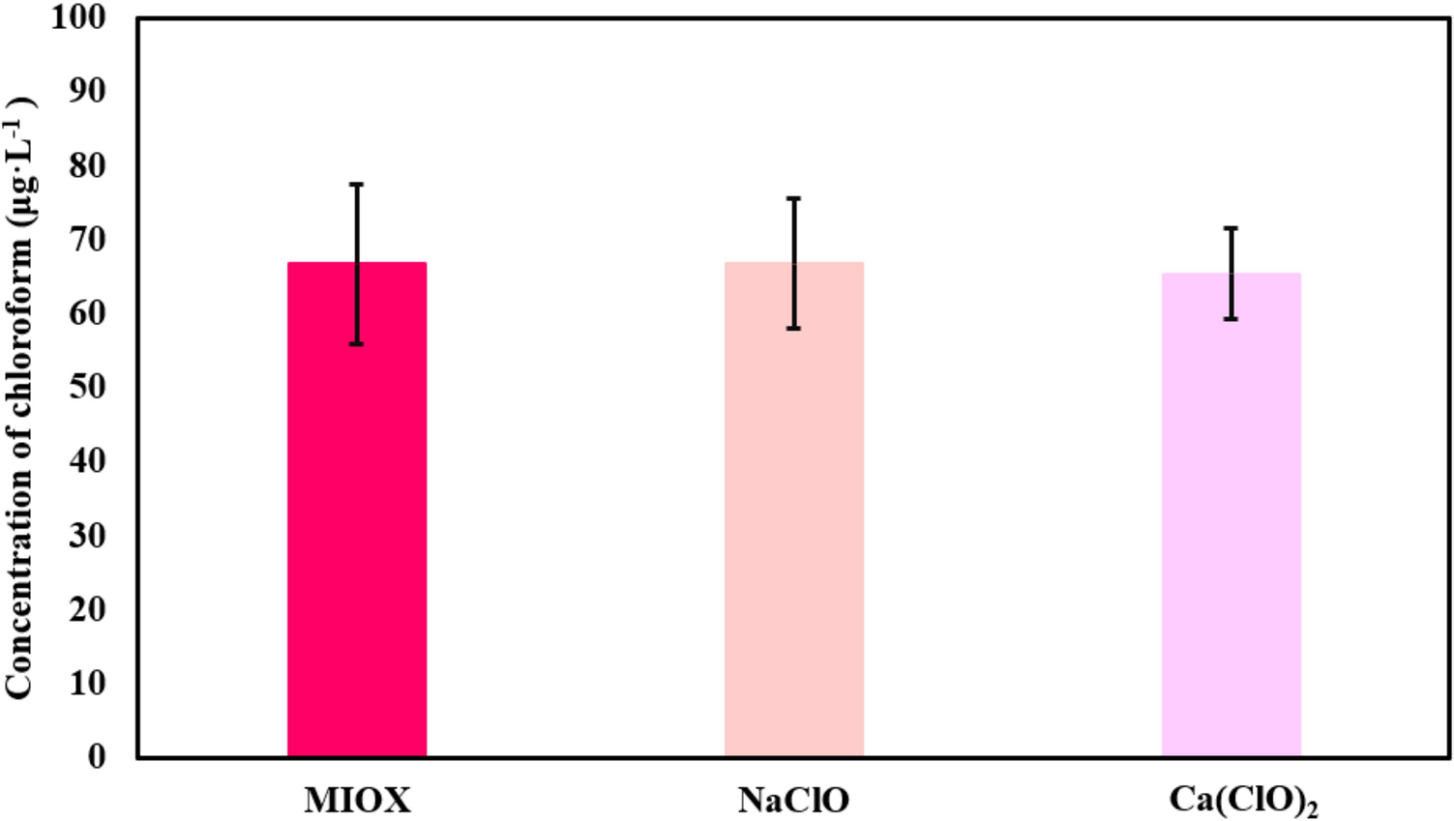
Concentration of chloroform in wastewater treated with NaClO, Ca(ClO)_2_, and MIOX

Figure 2 shows the concentration of THMs after disinfection with the hypochlorites NaClO and Ca(ClO)_2_, and MIOX.

The quantification of THM concentrations was performed by focusing on the main chemical species formed during chlorination, namely TCM, BDCM, DBCM, and TBM. No formation of DBCM, BDCM, and TBM was observed for the 2 mg Cl_2_·L^−1^ dose applied in the experiment. For the applied methodology, the detection limits determined for BDCM, DBCM, and TBM were 0.02, 0.02, and 0.01 μg·L^−1^, respectively. This result indicates that at lower dosages, chlorination exclusively favored the formation of TCM. Abreu Neto et al. (2025) evaluated the formation of THMs in wastewater chlorinated with high doses (55, 60, and 65 mg·L^−1^) of MIOX during breakpoint chlorination, under the same operational conditions used in the pretreatment of the effluent originating from the same wastewater treatment plant (ETE-Mangueira). The authors reported that TCM predominated at all MIOX dosages, with an average formation percentage ranging from 89.1% to 67.7%. The other brominated compounds (BDCM, DBCM, and TBM) showed lower contributions, ranging from 0.5% to 23.4% of the total THMs. These results suggest that, even with increasing MIOX dosage, TCM formation remains dominant, while the relative proportion of brominated THMs increases.

Thus, it is well established that high chlorine concentrations tend to favor the formation of brominated compounds, since chlorine oxidizes bromide (Br^−^) to HOBr, a species more reactive than HOCl in the generation of brominated THMs such as TBM (Gallard and Von Gunten 2002). In systems with excess chlorine, the production of oxidized halogen species increases, thereby enhancing the likelihood of halogenation reactions involving bromine (Gallard and Von Gunten 2002). However, this classical behavior reported in the literature was not observed in the present study, which can be attributed to the low dosage applied (2 mg Cl_2_·L^−1^). Under this operational condition, the oxidation of Br^−^ is limited, substantially reducing the potential for the formation of brominated THMs and resulting in the exclusive predominance of TCM observed in this work.

The formation of THMs occurs through a complex process involving multiple steps. During the chlorination of natural organic matter (NOM), the mechanistic pathways involve rapid electrophilic halogenation of phenolic compounds, forming halogenated intermediates (such as TCM), and slower reactions of enolizable carbonyl compounds, whose hydrolysis to form THMs can be catalytically enhanced by the hydroxide ion (OH⁻), generating THMs gradually (Gallard and Von Gunten 2002; Liu and Croué 2016). Secondary pathways involving non-aromatic enolized carbonyls also contribute, especially at later stages. Formation rates depend on both chlorine and precursor concentrations, explaining the observed THM profiles and providing insights for strategies to minimize their formation (Gallard and Von Gunten 2002). Figure 2 does not reveal differences in f TCM quantification for the different hypochlorites applied. The average concentrations found were 67 ± 8 and 67 ± 10 μg·L^−1^ of TCM for chlorination with NaClO and MIOX, respectively, and 65 ± 6 μg·L^−1^ of TCM for chlorination with Ca(ClO)_2_. These results are in accordance with resolution number 430/2011 of CONAMA (2011). This resolution establishes effluent discharge limits, specifically for quantifying 1000 μg·L^−1^ of TCM. Thus, the chlorinated effluent meets the requirements set by this regulation (CONAMA 2011).

The formation of THMs depends on several factors, such as the presence and composition of natural organic matter, pH, water temperature (which influences reaction rate), residual chlorine, contact time, and chlorine dosage (Ma et al. 2015). Thus, the predominance of TCM formation is due to the characteristics of the wastewater (Gallard and Von Gunten 2002; Rebelo et al. 2016; Kothe et al. 2024).

**Fig. 3:**
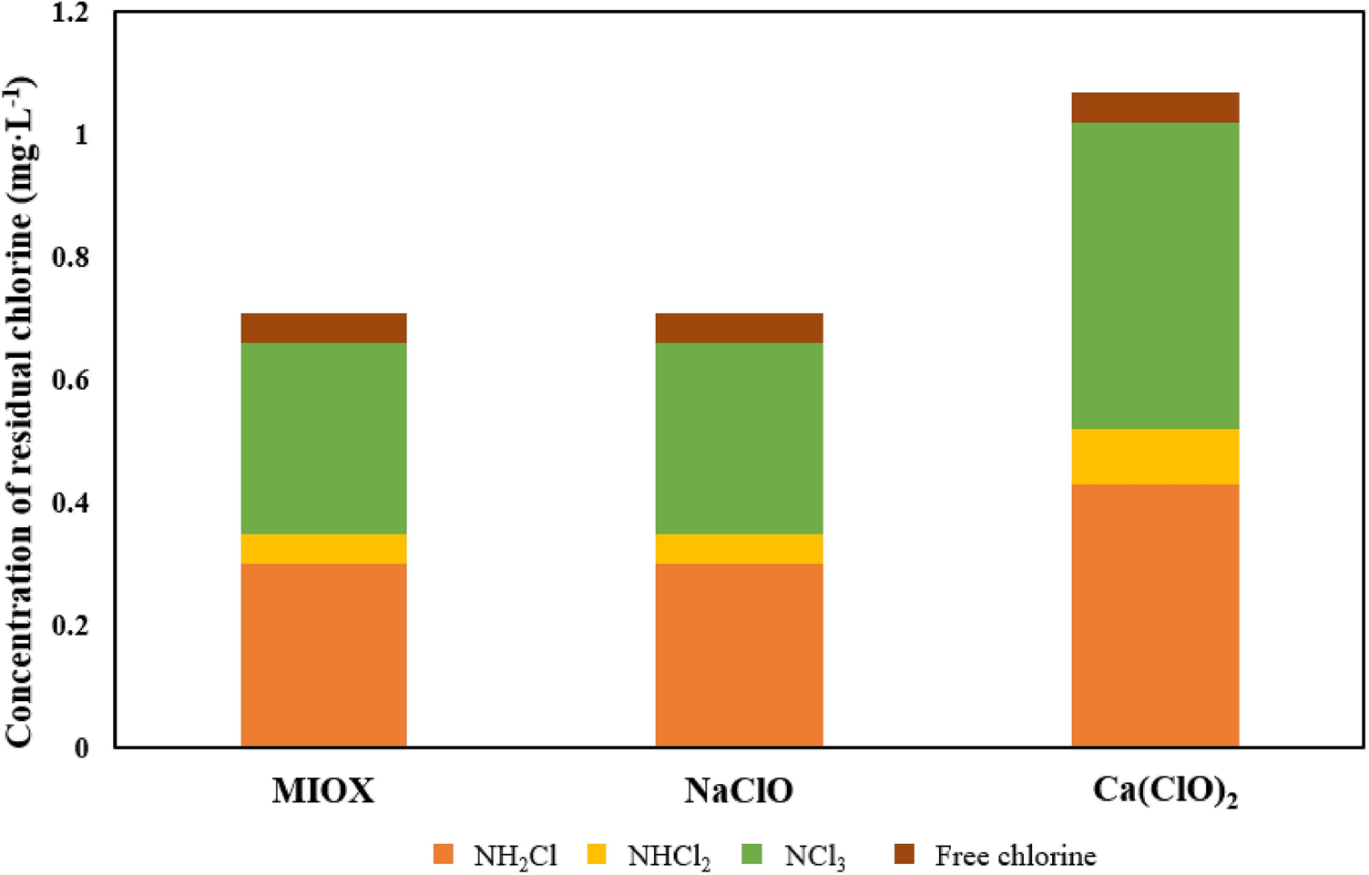
Residual chlorine (free chlorine and NH_2_Cl, NHCl_2_, and NCl_3_) after disinfection with NaClO, Ca(ClO)_2_, and MIOX

Another factor that can influence the formation of disinfection byproducts is the presence of nitrogen ammonia (NH_3_-N) in chlorinated wastewater (Du et al. 2017). Wang and colleagues (Wang et al. 2022b) found that the presence of NH_3_-N significantly reduced the formation of most DBPs by 82–100% (Wang et al. 2022b). Thus, the concentrations of chloramines, NH_2_Cl, NHCl_2_, and NCl_3_, as well as free chlorine, were also quantified (Fig. 3). This low concentration of TCM can also be explained by the chlorine mass (mg·L^−1^ Cl_2_) to NH_3_-N (mg·L^−1^ N) ratio (Cl_2_:N). The formation of DBPs increases with an increasing (Cl_2_:N) ratio. This behavior was observed by Zhang et al. (2011) when evaluating this ratio (Cl_2_:N) with a range of 4–11 during THM formation.

The estimated value of the NH_3_-N concentration in the present study was determined through theoretical calculations according to reaction (3) and the Henderson-Hasselbalch Eq. (4) (Skoog et al. 2021):

3 $${{NH}_{4}^{+}}_{(aq)}\leftrightharpoons {{NH}_{3}}_{(aq)}+ {{H}_{2}O}_{(aq)}$$

4 $$pH= {pK}_{a}-log\, ([HA]/[{A}^{-}])$$

where pKa = 9.25 and the values of pH and NH_4_^+^ were obtained experimentally.

In the present experiment, these (Cl_2_:N) ratios were < 1 for NaClO, Ca(ClO)_2_, and MIOX, which accounted for the low THM concentrations observed. Another factor that may have influenced this is reaction time, which is directly proportional to the increase in DBP concentration (Rebelo et al. 2016; Kothe et al. 2024). Figure 3 shows the concentration of residual chlorine (free chlorine and NH_2_Cl, NHCl_2_, and NCl_3_) after disinfection with NaClO, Ca(ClO)_2_, and MIOX.

After chlorination with 2 mg Cl_2_·L^−1^, a reduction in total chlorine was observed. The predominant species were NH_2_Cl and NCl_3_. The total chlorine concentrations were 0.71 mg·L^−1^ for NaClO and MIOX, and 1.07 mg·L^−1^ for Ca(ClO)_2_. For all three oxidants, the free chlorine (free chlorine) value was < 0.05 mg·L^−1^. The concentrations of NH_2_Cl, NHCl_2_, and NCl_3_ were, respectively, for NaClO: 0.30 ± 0.05, 0.05 ± 0.03, and 0.31 ± 0.05 mg·L^−1^; for Ca(ClO)_2_: 0.43 ± 0.14, 0.09 ± 0.08, and 0.50 ± 0.38 mg·L^−1^; and for MIOX: 0.30 ± 0.04, 0.05 ± 0.03, and 0.31 ± 0.04 mg·L^−1^.

Qiang and Adams et al. (2004) observed that NH_2_Cl was the dominant species when the Cl_2_:N ratio was ≤ 5, with pH between 6.5 and 8.5. The pH variation in the experiment was within the range observed by those authors, being 6.8 for NaClO, 6.7 for Ca(ClO)_2_, and 6.9 for MIOX. NCl_3_, due to its ability to undergo electron transfer reactions and electrophilic substitution, can also contribute to the formation of DBPs, such as chloroform (Soltermann et al. 2015). This formation occurs due to the reaction of NCl_3_ with certain compounds, such as 1,2-dihydroxybenzene, which can be generated from the reaction of organic compounds (such as humic and fulvic acids) with disinfectants (Soltermann et al. 2015).

Even though ammonia can react quickly with free chlorine and transform into chloramines during the chlorination process (Qiang and Adams 2004; Gleason et al. 2016), the reactivity of combined chlorine is much weaker than free chlorine, resulting in slower DBP formation during the reaction with organic matter in wastewater (Zhang et al. 2011). This reaction rate, along with other factors, may also have contributed to the low chloroform formation reported in Fig. 2.

Based on the results obtained and the literature, operational conditions can be optimized to minimize the formation of disinfection byproducts. Low doses of chlorinating agents (e.g., 2 mg Cl_2_·L^−1^) reduce THM formation, whereas higher doses favor the formation of brominated THMs (Abreu Neto et al. 2025), with TCM predominating at low doses of MIOX, NaClO, and Ca(ClO)_2_. This effect is associated with low Cl_2_:N ratios (< 1), as N-NH_4_^+^ promotes the formation of chloramines, which react more slowly with organic matter (Wang et al. 2022b; Zhang et al. 2011). In addition, pH values between 6.5 and 7.0 and shorter contact times also help limit the formation of disinfection byproducts (Kothe et al. 2024; Rebelo et al. 2016).

## Conclusions

The MIOX proved to be an alternative for the disinfection of anaerobic wastewater, showing performance equivalent to or superior in some microbiological and physicochemical parameters compared to conventional hypochlorites (NaClO and Ca(ClO)_2_). When evaluating the removal of total coliforms, the MIOX solution showed a greater reduction than Ca(ClO)2 or NaClO under the tested conditions. Microbiological results indicated greater efficiency in the removal of total coliforms for MIOX, with results showing a log reduction from 6.23 log_10_ MPN per 100 mL to 1.68 ± 0.13, 1.94 ± 0.08, and 2.53 ± 0.39 log_10_ MPN per 100 mL for Ca(ClO)_2_, NaClO, and MIOX, respectively. In the case of *E. coli*, all oxidizing agents showed similar effectiveness.

Among the physicochemical parameters analyzed, the increase in electrical conductivity was particularly notable. Conductivity values increased from 0.2 μS·cm^−1^ to 587, 597, and 810 μS·cm^−1^ for NaClO, Ca(ClO)_2_, and MIOX, respectively. This increase indicates a higher concentration of dissolved ionic species, which may influence the effectiveness of oxidative treatments in aqueous matrices. The increase in Na⁺ ion concentration observed in the treated effluent should be especially considered among the effluent quality parameters in future studies involving the application of MIOX technology.

Regarding THM_4_ formation, at the low dosage used (2 mg Cl_2_·L^−1^), chlorination predominantly favored the formation of TCM, with no detection of BDCM, DBCM, or TBM, whose method detection limits are 0.02, 0.02, and 0.01 μg·L^−1^, respectively.

## Recommendations and future research suggestions

It is recommended that future research investigate the chemical interactions among the oxidizing agents Ca(ClO)2, NaClO, and MIOX in the formation of other DBPs, including haloacetic acids, which are common chlorination products. Examples include monochloroacetic acid, dichloroacetic acid, trichloroacetic acid, and bromoacetic acids. Another parameter that should be quantified is total organic carbon (TOC), to more accurately assess the influence of dissolved organic matter on DBP formation and Br^−^ and I^−^ ions. Furthermore, it is suggested that the toxicity of the MIOX solution be compared with that of commercial hypochlorites (Ca(ClO)_2_, NaClO). It is recommended that future studies evaluate and compare disinfection byproduct formation and microbial reduction using alternative methods, such as ozonation and chlorine dioxide, in relation to MIOX chlorination. Finally, it is recommended to assess the technical and economic feasibility of large-scale MIOX applications through pilot studies in wastewater treatment plants. This analysis will allow for the estimation of operational costs, equipment durability, and system effectiveness over the long term, supporting its implementation in real-world scenarios. In this way, it will be possible to develop more efficient and sustainable wastewater treatment strategies.

## Supplementary Information

Below is the link to the electronic supplementary material.

Supplementary file1 (DOCX 44 KB)

## Data Availability

All data generated or analyzed during this study are included in this published article.
