# Supplementary material for: Disinfection performance of mixed oxidant, NaClO, and Ca(ClO)2 in anaerobic effluents: effects on microbial quality, physicochemical properties, and trihalomethane formation
Source: Environ Sci Pollut Res Int. 2026 Aug 3;33(25):12774–86. doi: 10.1007/s11356-026-38060-9 (PMC13476181; doi:10.1007/s11356-026-38060-9)
Supplement: Supplementary file 1 [file 11356_2026_38060_MOESM1_ESM.docx]

**Supplementary Information (SI)**

Determination of contact time and working dosage

The optimal contact time and dosage were determined based on the results shown in Table A1, which present the effects of combining contact times (10, 15, and 20 min) and dosages (1.0, 1.5, and 2.0 mg·L^-1^). The results obtained represent, respectively, the actions of the commercial hypochlorites Ca(ClO)2 and NaClO, and the MIOX solution, as disinfection agents for anaerobic effluents. As a response, the efficacy in reducing the concentration of total coliforms and *E. coli* was measured. In addition to the two parameters, contact time and dosage, additional aspects are presented in Table1, including total residual chlorine (TRC) concentration. The initial concentration of total coliforms was 1732·10^3^ MPN·100 mL^-1^, and of *E. coli* was 816·10^3^ MPN·100 mL^-1^. The entire experiment was conducted at 24.0 ± 0.3 °C.

Table A1 Display of the contact time (min), dosage (mg·L^-1^), and the concentrations of total coliforms, E. coli (MPN·100 mL^-1^), and total residual chlorine for the disinfection process using MIOX, NaClO e Ca(ClO)_2_.

|  | **CT (min)** | **Dosage (mg***·***L^-1^)** | **TC**  **(MPN***·***100** **mL^-1^)** | ***E. coli***  **(MPN***·***100** **mL^-1^)** | **TRC (mg***·***L^-1^)** |
| --- | --- | --- | --- | --- | --- |
| **MIOX** | 10 | 1.0 | 2.41·10^4^ | 1.46 ·10^3^ | 0.25 |
|  | 15 | 1.0 | 2.41·10^4^ | 9.30 ·10 | 0.21 |
|  | 20 | 1.0 | 1.98 ·10^4^ | 4.00 ·10 | 0.18 |
|  | 10 | 1.5 | 5.04 ·10^4^ | 2.00 ·10^3^ | 1.37 |
|  | 15 | 1.5 | 3.10 ·10^3^ | <1.00 ·10^3^ | 1.26 |
|  | 20 | 1.5 | <1.00 ·10^3^ | <1.00 ·10^3^ | 1.23 |
|  | 10 | 2.0 | <1.00 ·10^3^ | <1.00 ·10^3^ | 1.94 |
|  | 15 | 2.0 | <1.00 ·10^3^ | <1.00 ·10^3^ | 1.91 |
|  | 20 | 2.0 | <1.00 ·10^3^ | <1.00 ·10^3^ | 1.87 |
| **NaClO** | 10 | 1.0 | 2.41·10^4^ | 4.88 ·10^3^ | 0.41 |
|  | 15 | 1.0 | 2.41·10^4^ | 1.90 ·10^2^ | 0.32 |
|  | 20 | 1.0 | 2.41·10^4^ | 9.40 ·10 | 0.21 |
|  | 10 | 1.5 | 1.04 ·10^6^ | 4.06 ·10^4^ | 1.02 |
|  | 15 | 1.5 | 1.51·10^5^ | 3.00 ·10^3^ | 0.90 |
|  | 20 | 1.5 | 3.13·10^4^ | <1.00 ·10^3^ | 0.75 |
|  | 10 | 2.0 | 2.00 ·10^3^ | <1.00 ·10^3^ | 1.98 |
|  | 15 | 2.0 | <1.00 ·10^3^ | <1.00 ·10^3^ | 1.93 |
|  | 20 | 2.0 | <1.00 ·10^3^ | <1.00 ·10^3^ | 1.86 |
| **Ca(ClO)_2_** | 10 | 1.0 | 2.41·10^4^ | 1.73 ·10^4^ | <0.15 |
|  | 15 | 1.0 | 2.41·10^4^ | 4.98 ·10^2^ | <0.15 |
|  | 20 | 1.0 | 2.41·10^4^ | 9.50 ·10 | <0.15 |
|  | 10 | 1.5 | 1.98 ·10^6^ | 1.56 ·10^4^ | 0.68 |
|  | 15 | 1.5 | 1.57 ·10^5^ | 4.10 ·10^3^ | 0.60 |
|  | 20 | 1.5 | 1.78 ·10^5^ | 3.00 ·10^3^ | 0.48 |
|  | 10 | 2.0 | 1·10^3^ | <1·10^3^ | 1.97 |
|  | 15 | 2.0 | <1·10^3^ | <1·10^3^ | 1.92 |
|  | 20 | 2.0 | <1·10^3^ | <1·10^3^ | 1.88 |

CT: contact time; TC: total coliforms; TRC: total residual chlorine

As shown in Table A1, the TRC concentrations corresponded to the dosages applied in the study. In this way, it was possible to identify the lower dosages and shorter contact times that effectively reduced the microbiological concentration of total coliforms and *E. coli*. It can also be observed that the MIOX solution showed the greatest reduction in the microbiological load of total coliforms and *E. coli* at a dosage of 1.5 mg·L^-1^. The concentrations obtained were <1.00 × 10^3^ MPN per 100 mL for both parameters. This result showed a notable reduction compared with the commercially available hypochlorites, Ca(ClO)_2_ and NaClO, with a similar effect observed only at a dosage of 2 mg·L^-1^. Thus, this analysis is promising for the water and wastewater industry, as lower concentrations will reduce operational costs and shorter contact times will optimize treatment processes. A more in-depth discussion will be addressed in the microbiological section ahead.

Therefore, the disinfection tests used a dosage of 2.0 mg·L^-1^ and a contact time of 20 min. This choice was made to standardize experimental parameters and ensure reliability across trials.
